# International electronic health record-derived post-acute sequelae profiles of COVID-19 patients

**DOI:** 10.1038/s41746-022-00623-8

**Published:** 2022-06-29

**Authors:** Harrison G. Zhang, Arianna Dagliati, Zahra Shakeri Hossein Abad, Xin Xiong, Clara-Lea Bonzel, Zongqi Xia, Bryce W. Q. Tan, Paul Avillach, Gabriel A. Brat, Chuan Hong, Michele Morris, Shyam Visweswaran, Lav P. Patel, Alba Gutiérrez-Sacristán, David A. Hanauer, John H. Holmes, Malarkodi Jebathilagam Samayamuthu, Florence T. Bourgeois, Sehi L’Yi, Sarah E. Maidlow, Bertrand Moal, Shawn N. Murphy, Zachary H. Strasser, Antoine Neuraz, Kee Yuan Ngiam, Ne Hooi Will Loh, Gilbert S. Omenn, Andrea Prunotto, Lauren A. Dalvin, Jeffrey G. Klann, Petra Schubert, Fernando J. Sanz Vidorreta, Vincent Benoit, Guillaume Verdy, Ramakanth Kavuluru, Hossein Estiri, Yuan Luo, Alberto Malovini, Valentina Tibollo, Riccardo Bellazzi, Kelly Cho, Yuk-Lam Ho, Amelia L. M. Tan, Byorn W. L. Tan, Nils Gehlenborg, Sara Lozano-Zahonero, Vianney Jouhet, Luca Chiovato, Bruce J. Aronow, Emma M. S. Toh, Wei Gen Scott Wong, Sara Pizzimenti, Kavishwar B. Wagholikar, Mauro Bucalo, The Consortium for Clinical Characterization of COVID-19 by EHR (4CE), Tianxi Cai, Andrew M. South, Isaac S. Kohane, Griffin M. Weber

**Affiliations:** 1 Department of Biomedical Informatics, Harvard Medical School, Boston, MA USA; 2 Department of Electrical Computer and Biomedical Engineering, University of Pavia, Pavia, Italy; 3 Department of Biostatistics, Harvard T.H. Chan School of Public Health, Boston, MA USA; 4 Department of Neurology, University of Pittsburgh, Pittsburgh, PA USA; 5 Department of Medicine, National University Hospital, Singapore, Singapore; 6 Department of Biostatistics and Bioinformatics, Duke University, Durham, NC USA; 7 Department of Biomedical Informatics, University of Pittsburgh, Pittsburgh, PA USA; 8 Department of Internal Medicine, Division of Medical Informatics, University Of Kansas Medical Center, Kansas City, MO USA; 9 Department of Learning Health Sciences, University of Michigan Medical School, Ann Arbor, MI USA; 10 Department of Biostatistics, Epidemiology, and Informatics, University of Pennsylvania Perelman School of Medicine, Philadelphia, PA USA; 11 Institute for Biomedical Informatics, University of Pennsylvania Perelman School of Medicine, Philadelphia, PA USA; 12 Department of Pediatrics, Harvard Medical School, Boston, MA USA; 13 Michigan Institute for Clinical and Health Research (MICHR) Informatics, University of Michigan, Ann Arbor, MI USA; 14 IAM unit, Bordeaux University Hospital, Bordeaux, France; 15 Department of Neurology, Massachusetts General Hospital, Boston, MA USA; 16 Department of Medicine, Massachusetts General Hospital, Boston, MA USA; 17 Department of biomedical informatics, Hôpital Necker-Enfants Malade, Assistance Publique Hôpitaux de Paris (APHP), University of Paris, Paris, France; 18 Department of Biomedical informatics, WiSDM, National University Health Systems Singapore, Singapore, Singapore; 19 Department of Anaesthesia, National University Health Systems Singapore, Singapore, Singapore; 20 Department of Computational Medicine & Bioinformatics, Internal Medicine, Human Genetics, and School of Public Health, University of Michigan, Ann Arbor, MI USA; 21 Institute of Medical Biometry and Statistics, Faculty of Medicine and Medical Center, University of Freiburg, Freiburg, Germany; 22 Department of Ophthalmology, Mayo Clinic, Rochester, NY USA; 23 Massachusetts Veterans Epidemiology Research and Information Center (MAVERIC), VA Boston Healthcare System, Boston, MA USA; 24 Department of Medicine, David Geffen School of Medicine at UCLA, Los Angeles, CA USA; 25 IT Department, Innovation & Data, APHP Greater Paris University Hospital, Paris, France; 26 Division of Biomedical Informatics (Department of Internal Medicine), University of Kentucky, Lexington, KY USA; 27 Department of Preventive Medicine, Northwestern University, Chicago, IL USA; 28 Laboratory of Informatics and Systems Engineering for Clinical Research, Istituti Clinici Scientifici Maugeri SpA SB IRCCS, Pavia, Italy; 29 Department of Electrical, Computer and Biomedical Engineering, University of Pavia, Pavia, Italy; 30 Population Health and Data Science, VA Boston Healthcare System, Boston, MA USA; 31 IAM unit, INSERM Bordeaux Population Health ERIAS TEAM, Bordeaux University Hospital / ERIAS - Inserm, U1219 BPH Bordeaux, France; 32 Unit of Internal Medicine and Endocrinology, Istituti Clinici Scientifici Maugeri SpA SB IRCCS, Pavia, Italy; 33 Departments of Biomedical Informatics, Pediatrics, Cincinnati Children’s Hospital Medical Center, University of Cincinnati, Cincinnati, OH USA; 34 Yong Loo Lin School of Medicine, National University of Singapore, Singapore, Singapore; 35 Department of Medicine, National University Health Systems Singapore, Singapore, Singapore; 36 Scientific Direction, IRCCS Ca’ Granda Ospedale Maggiore Policlinico di Milano, Milan, Italy; 37 BIOMERIS (BIOMedical Research Informatics Solutions), Pavia, Italy; 38 Department of Pediatrics-Section of Nephrology, Brenner Children’s, Wake Forest School of Medicine, Winston Salem, NC USA

**Keywords:** Outcomes research, Databases, Viral infection

## Abstract

The risk profiles of post-acute sequelae of COVID-19 (PASC) have not been well characterized in multi-national settings with appropriate controls. We leveraged electronic health record (EHR) data from 277 international hospitals representing 414,602 patients with COVID-19, 2.3 million control patients without COVID-19 in the inpatient and outpatient settings, and over 221 million diagnosis codes to systematically identify new-onset conditions enriched among patients with COVID-19 during the post-acute period. Compared to inpatient controls, inpatient COVID-19 cases were at significant risk for angina pectoris (RR 1.30, 95% CI 1.09–1.55), heart failure (RR 1.22, 95% CI 1.10–1.35), cognitive dysfunctions (RR 1.18, 95% CI 1.07–1.31), and fatigue (RR 1.18, 95% CI 1.07–1.30). Relative to outpatient controls, outpatient COVID-19 cases were at risk for pulmonary embolism (RR 2.10, 95% CI 1.58–2.76), venous embolism (RR 1.34, 95% CI 1.17–1.54), atrial fibrillation (RR 1.30, 95% CI 1.13–1.50), type 2 diabetes (RR 1.26, 95% CI 1.16–1.36) and vitamin D deficiency (RR 1.19, 95% CI 1.09–1.30). Outpatient COVID-19 cases were also at risk for loss of smell and taste (RR 2.42, 95% CI 1.90–3.06), inflammatory neuropathy (RR 1.66, 95% CI 1.21–2.27), and cognitive dysfunction (RR 1.18, 95% CI 1.04–1.33). The incidence of post-acute cardiovascular and pulmonary conditions decreased across time among inpatient cases while the incidence of cardiovascular, digestive, and metabolic conditions increased among outpatient cases. Our study, based on a federated international network, systematically identified robust conditions associated with PASC compared to control groups, underscoring the multifaceted cardiovascular and neurological phenotype profiles of PASC.

## Introduction

There is growing evidence that long-lasting, post-acute sequelae of COVID-19 (PASC) develop after severe acute respiratory syndrome coronavirus 2 (SARS-CoV-2) infection. Previous studies have reported that PASC, or long-COVID symptoms, may include fatigue, shortness of breath, pain, difficulty concentrating, and depression^1–4^. These symptoms may persist for months after the initial infection even in patients who do not develop severe disease^5–10^. Despite the high prevalence of these persistent symptoms, there is a substantial lag in knowledge about the spectrum of complications arising from the initial infection. A greater understanding of PASC phenotypes and risk factors is needed to develop evidence-based evaluation and management guidelines.

The current PASC literature consists of single-center studies based on follow-up in-person or telephone surveys, which have had a limited scope, power, and generalizability^2,3,11^. Recently, large-scale, multicenter, electronic health record (EHR) studies have been reported, which may improve the generalizability and understanding of PASC to inform public health experts, health workers, and patients of the risk of long-term complications from SARS-CoV-2 infection^12–15^. However, there have been limited coordinated attempts at an international level aiming to leverage widely available EHR data to systematically study PASC as few of the current multicenter studies include an international cohort^12–16^. Further, apart from small sample sizes, many multicenter studies are limited in their focus on PASC relating to specific body systems. Lastly, few existing multicenter studies consider appropriate control groups and none of the current studies exploit disease trajectories of progression in specific time windows, nor in calendar time^16–19^.

In this study, we extracted, consolidated, harmonized, and analyzed EHR data from an international cohort of patients from the healthcare systems participating in the Consortium for Clinical Characterization of COVID-19 by EHR (4CE). The 4CE Consortium is a research collaborative across seven countries that uses EHR data in a federated manner to study the epidemiology and clinical course of COVID-19^20,21^. The 4CE network of researchers manually ran database queries returning only aggregate counts and statistics on data representative of 414,602 patients infected with SARS-CoV-2 and 2.3 million controls with a negative test for SARS-CoV-2 infection from 18 healthcare systems. The results were uploaded to a central site for analysis.

We considered patients who were hospitalized at the time of SARS-CoV-2 infection (herein referred to as inpatient COVID-19 cases) and patients who were not hospitalized during SARS-CoV-2 infection (outpatient COVID-19 cases). We defined the acute stage as within 29 days after infection, the mid-stage post-acute period as 30 to 89 after initial infection, and the late-stage post-acute period as 90+ days after initial infection.

We aimed to (1) establish the feasibility and interoperability of extracting EHR data in a federated manner for studying PASC; (2) use codified EHR data to identify incident conditions of higher risk in inpatient COVID-19 cases compared to controls; (3) identify incident conditions of higher risk in outpatient COVID-19 cases compared to controls; and (4) examine temporal patterns in cumulative incidence of conditions during the mid-stage post-acute period based on the calendar quarter in which patients were infected with SARS-CoV-2.

## Results

### Description of the study population

Data for this study were contributed by 277 hospitals, with 42 in France, 1 in Germany, 4 in Italy, 1 in Singapore, and 228 in the US. The study population consists of a total of 75,232 inpatient COVID-19 cases, 339,370 outpatient COVID-19 cases, 505,055 inpatient controls, and 1,825,473 outpatient controls who were tested for SARS-CoV-2 between the first quarter of 2020 (2020-Q1) through the first quarter of 2021 (2021-Q1).

**Fig. 1:**
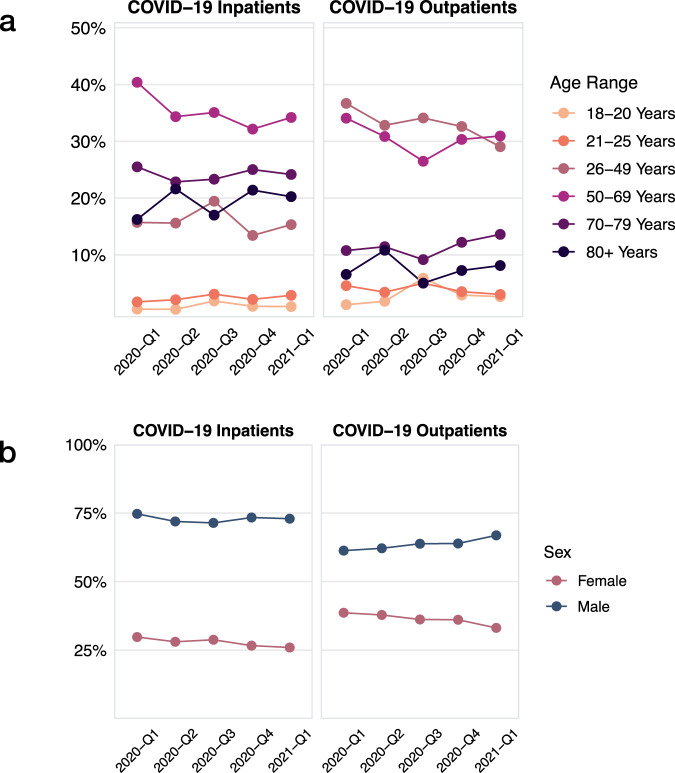
Demographic trends for age and sex across calendar time in the study population. **a** Age trends among inpatient COVID-19 cases and outpatient COVID-19 cases. **b** Sex trends among inpatient COVID-19 cases and outpatient COVID-19 cases.

We report the demographic characteristics of patients with COVID-19 over different periods of the pandemic in Fig. 1. Comparing inpatient COVID-19 cases admitted in 2020-Q1 to 2021-Q1, the proportion of inpatient COVID-19 cases aged 50–69 years decreased (Δ = −7.83%, *P* = 0.001). Among outpatient COVID-19 cases, the proportion of patients aged 26–49 years decreased (Δ = −7.97%, *P* < 0.001) while the proportion aged 70–79 years increased (Δ = 4.57%, *P* = 0.004). Demographic profiles for age and sex among inpatient and outpatient COVID-19 cases and their corresponding controls were comparable (Table 1 and Supplementary Fig. 1).

**Table 1:** Proportion of subgroups (95% confidence intervals) of age and sex among inpatient and outpatient COVID-19 cases and corresponding control cohorts.

	COVID-19 inpatients		Controls		COVID-19 outpatients		Controls	
Age								
18 to 20	0.01	[0.01, 0.01]	0.01	[0.01, 0.02]	0.03	[0.02, 0.04]^a^	0.01	[0.01, 0.01]^a^
21 to 25	0.02	[0.02, 0.03]	0.01	[0.01, 0.02]	0.04	[0.03, 0.05]	0.03	[0.03, 0.04]
26 to 49	0.16	[0.14, 0.18]	0.18	[0.16, 0.20]	0.33	[0.31, 0.34]	0.32	[0.31, 0.33]
50 to 69	0.35	[0.34, 0.36]	0.33	[0.32, 0.35]	0.30	[0.29, 0.32]	0.32	[0.30, 0.33]
70 to 79	0.24	[0.22, 0.26]	0.22	[0.20, 0.25]	0.11	[0.10, 0.13]	0.13	[0.11, 0.14]
80 plus	0.19	[0.18, 0.21]^a^	0.15	[0.14, 0.16]^a^	0.07	[0.06, 0.09]	0.05	[0.05, 0.06]
Sex								
Female	0.26	(0.22, 0.31)	0.24	(0.20, 0.30)	0.36	(0.31, 0.42)	0.41	(0.36, 0.46)
Male	0.74	(0.70, 0.79)	0.76	(0.70, 0.80)	0.64	(0.58, 0.69)	0.59	(0.54, 0.64)

^a^Statistically significant difference in comparison to controls.

### Baseline prevalence and acute period incidence of conditions

**Fig. 2:**
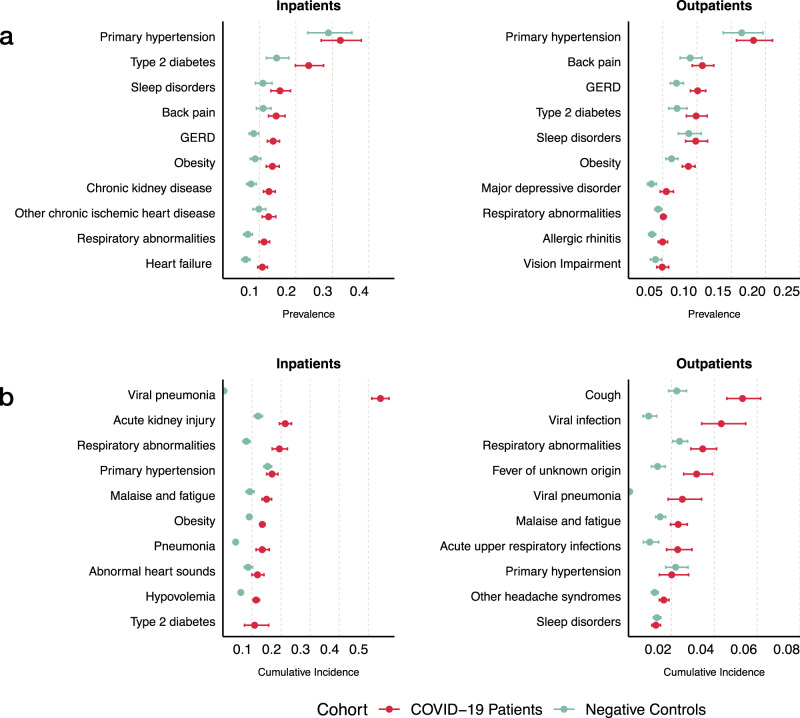
Clinical characteristics of the study population. **a** Most prevalent preexisting conditions in COVID-19 patients compared to the controls stratified by hospitalization status. **b** Conditions with the highest cumulative incidence during the acute stage up to 29 days after initial infection.

Our dataset encompassed over 920 medical conditions as defined by phenotype code (PheCode) from the Phenome-wide association studies (PheWAS) catalog of phenotypes^22,23^. When compared to inpatient controls, inpatient COVID-19 cases had a higher baseline prevalence of type 2 diabetes, gastroesophageal disease, obesity, chronic kidney disease, respiratory abnormalities, and heart failure (Fig. 2a). Among inpatient COVID-19 cases, conditions with the highest cumulative incidence during the acute stage included viral pneumonia, acute kidney injury, respiratory abnormalities, primary hypertension, malaise, and fatigue (Fig. 2b). When compared to inpatient controls, inpatient COVID-19 cases had a higher cumulative incidence of viral pneumonia, respiratory abnormalities, pneumonia, malaise, fatigue, acute kidney injury, and hypovolemia.

When compared to outpatient controls, outpatient COVID-19 cases had a higher baseline prevalence of gastroesophageal disease, obesity, and major depressive disorder (Fig. 2a). Conditions with the highest cumulative incidence in the acute stage included cough, viral infection, respiratory abnormalities, fever, and viral pneumonia (Fig. 2b). As expected, outpatient COVID-19 cases had a higher cumulative incidence of viral infection, viral pneumonia, cough, respiratory abnormalities, acute upper respiratory infections, fever of unknown origin, malaise, and fatigue compared to outpatient controls.

### Incident high-risk conditions at mid and late-stage post-acute periods in inpatient COVID-19 cases

**Fig. 3:**
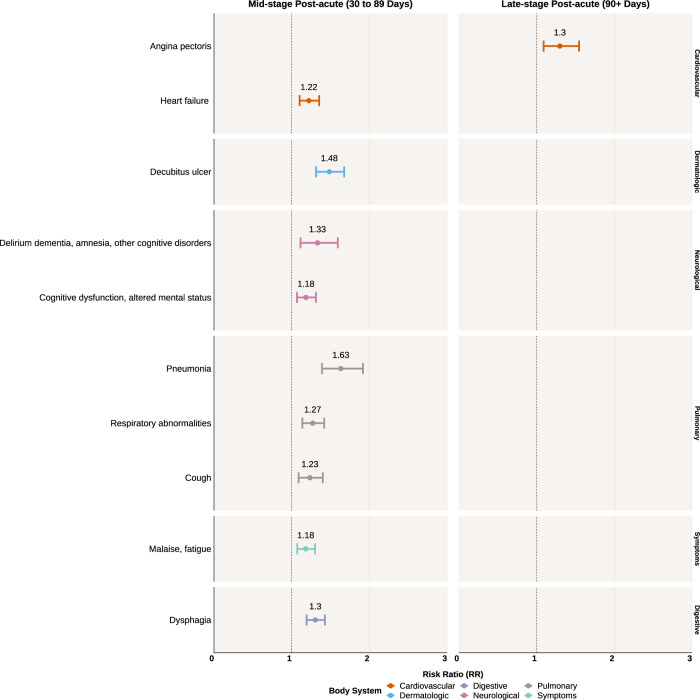
Statistically significant risk ratios and their 95% confidence intervals of health conditions in the inpatient COVID-19 cohort compared to the control inpatient cohort. Left panel shows diseases of increased risk at the mid-stage post-acute period (30 to 89 days after initial infection) among the inpatient COVID-19 cohort. Right panel shows diseases of increased risk at the latestage post-acute period (90+ days after initial infection). *P* values were corrected for multiple comparisons with a 5% false discovery rate.

Inpatient COVID-19 cases were at significantly higher risk for incident cardiovascular, neurological, and pulmonary conditions compared to inpatient controls at the mid-stage post-acute period after correction for multiple comparisons (Fig. 3). There was an increased risk for heart failure (RR 1.22, 95% CI 1.10–1.35) and the pulmonary conditions of pneumonia (RR 1.63, 95% CI 1.39–1.92), respiratory abnormalities (RR 1.27, 95% CI 1.14–1.42), and cough (RR 1.23, 95% CI 1.09–1.40). Neurological conditions of increased risk included delirium dementia, amnesia, and other cognitive disorders (RR 1.33, 95% CI 1.11–1.59), and cognitive dysfunction or altered mental status (RR 1.18, 95% CI 1.07–1.31). Inpatient COVID-19 cases also experienced a greater risk for symptoms of malaise and fatigue (RR 1.18, 95% CI 1.07–1.30).

During the late-stage period, inpatient COVID-19 cases had an increased risk for angina pectoris (RR 1.3, 95% CI 1.09–1.55). There were no conditions which persisted from the mid-stage to the late-stage period. We use the term “persistent” to reflect an association being statistically significant for both mid- and late-stage post-acute periods.

### Incident high-risk conditions at mid and late-stage post-acute periods in outpatient COVID-19 cases

**Fig. 4:**
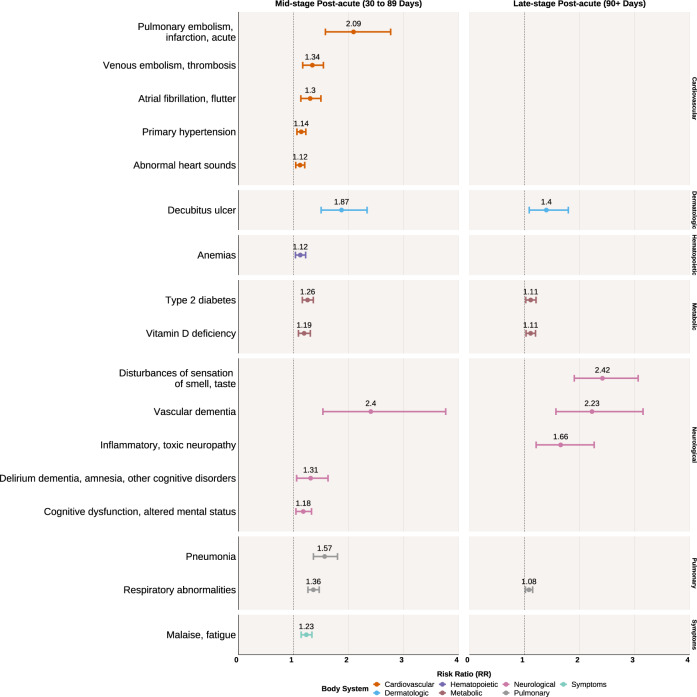
Statistically significant risk ratios with 95% confidence intervals of health conditions in the outpatient COVID-19 cohort compared to the control outpatient cohort. Left panel shows diseases of increased risk at the mid-stage post-acute period (30 to 89 days after initial infection) among the outpatient COVID-19 cohort. Right panel shows diseases of increased risk at the late-stage post-acute period (90+ days after initial infection). *P* values were corrected for multiple comparisons with a 5% false discovery rate.

Outpatient COVID-19 cases were at significantly higher risk for incident cardiovascular, metabolic, neurological, and pulmonary conditions compared to outpatient controls at the mid-stage post-acute period (Fig. 4). There was a greater risk for embolic diseases such as acute pulmonary embolism and infarction (RR 2.09, 95% CI 1.58–2.76) and venous embolism and thrombosis (RR 1.34, 95% CI 1.17–1.54). Additionally, there was an increased risk for atrial fibrillation and flutter (RR 1.30, 95% CI 1.13–1.50) and primary hypertension (RR 1.14, 95% CI 1.06–1.22). Metabolic conditions with increased risk included type 2 diabetes (RR 1.26, 95% CI 1.16–1.36) and vitamin D deficiency (RR 1.19, 95% CI 1.09–1.30). Outpatient COVID-19 cases were also at increased risk for neurological conditions including vascular dementia (RR 2.40, 95% CI 1.53–3.76), derulium dementia, amnesia, and other cognitive disorders (RR 1.31, 95% CI 1.06–1.63), and cognitive dysfunction or altered mental status (RR 1.18, 95% CI 1.04–1.33). There was also an increased risk for pneumonia (RR 1.57, 95% CI 1.36–1.80) as well as malaise and fatigue (RR 1.23, 95% CI 1.14–1.34).

During the late-stage period, when compared to outpatient controls, outpatient COVID-19 cases had a persistently increased risk for decubitus ulcers (RR 1.40, 95% CI 1.09–1.80), type 2 diabetes (RR 1.11, 95% CI 1.02–1.21), vitamin D deficiency (RR 1.11, 95% CI 1.03–1.20), vascular dementia (RR 2.23, 95% CI 1.57–3.15), and respiratory abnormalities (RR 1.08, 95% CI 1.02–1.15), though the magnitude of these estimates were attenuated slightly compared to the mid-stage period. Conditions unique to the late-stage period included disturbances of sensation of smell and taste (RR 2.42, 95% CI 1.90–3.06) and inflammatory or toxic neuropathy (RR 1.66, 95% CI 1.21–2.27).

### Differences in PASC conditions between inpatient and outpatient COVID-19 cases in the mid-stage period

Inpatient COVID-19 cases were at greater risk for dysphagia (relative RR 1.46, 95% CI 1.16–1.84) compared to outpatient COVID-19 cases. No other phenotypes were significant after correction for multiple comparisons.

### Changes in PASC cumulative incidence by calendar quarter

**Fig. 5:**
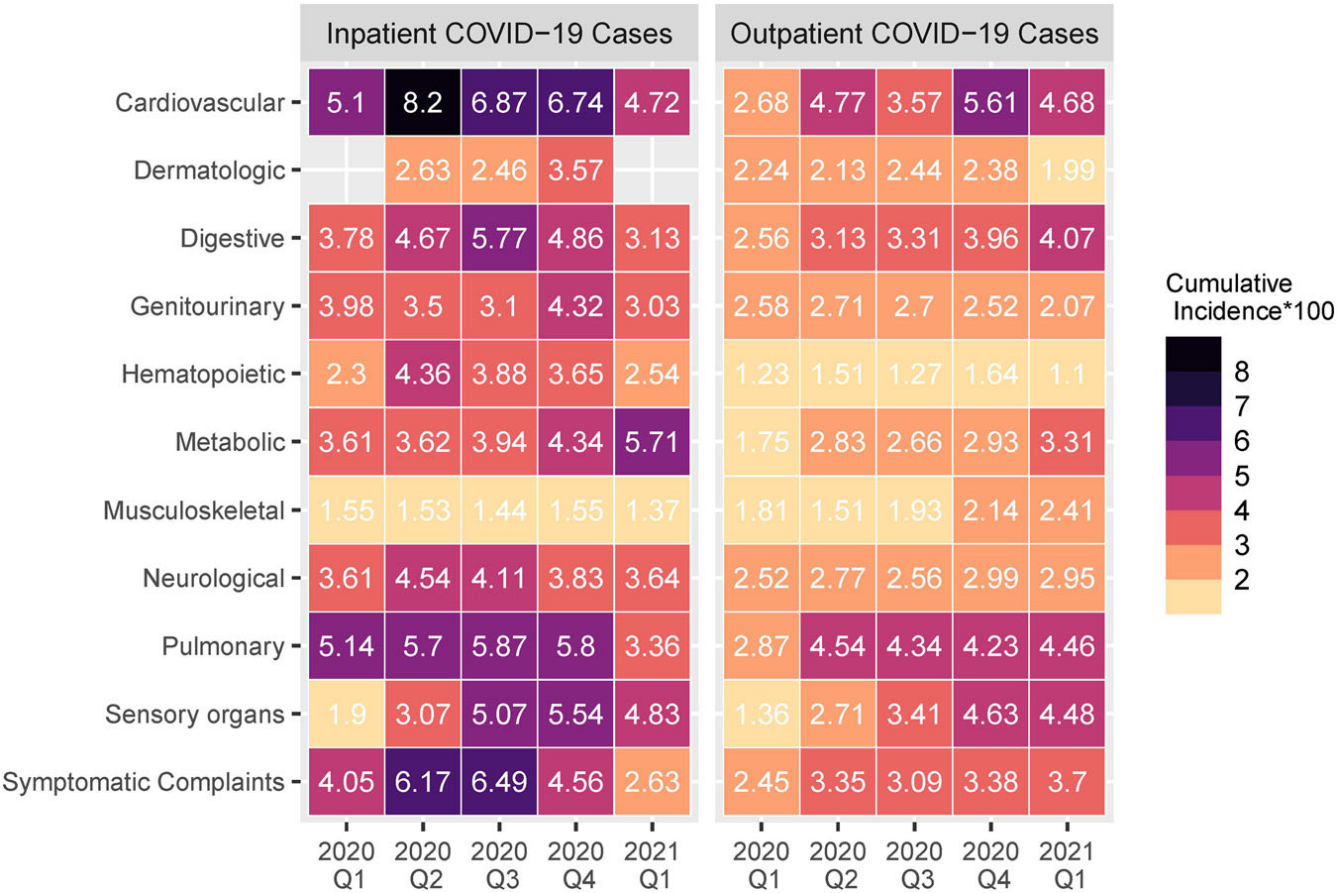
Cumulative incidence of various conditions at the mid-stage post-acute period (30 to 89 days after initial infection) by the calendar quarter of their initial infection date. Left panel shows cumulative incidence among inpatient COVID-19 cases. Right panel shows cumulative incidence among outpatient COVID-19 cases.

We examined temporal changes in the cumulative incidence of conditions over the pandemic grouped by organ system for inpatient and outpatient COVID-19 cases at the mid-stage period, based on calendar quarters (Fig. 5). Among the inpatient COVID-19 cases, the incidence of cardiovascular and pulmonary conditions as well as symptomatic complaints declined across time, while the incidence of metabolic conditions increased. Among the outpatient COVID-19 cases, the incidence of cardiovascular, digestive, metabolic, and sensory organ conditions increased while the other conditions remained relatively constant.

## Discussion

We leveraged the existing healthcare system infrastructure to collect and analyze aggregated patient-level EHR data from patients with COVID-19 and control patients across five countries to begin to better define PASC phenotypes using a well-validated common data model. In addition to the expected higher incidence of pulmonary conditions as well as malaise and fatigue, we observed that hospitalized patients with COVID-19 had a greater risk of new cardiovascular and neurological conditions when compared to inpatient controls. Additionally, patients diagnosed with COVID-19 in the outpatient setting had a greater risk of new embolic and thrombotic conditions, hypertension, atrial fibrillation, neurological conditions, and disorders of smell and taste. Our federated approach is in contrast to prior efforts to characterize PASC phenotypes using a prevalence of symptoms and diagnoses, which, in the absence of appropriate non-COVID-19 patient control groups, could not be meaningfully interpreted, and is in contrast to multicenter centralized analyses with smaller sample sizes^19^.

This study used a federated approach, in which standardized and straightforward database queries were distributed to sites to run locally on their EHR data, and only aggregate counts and statistics were shared externally. This approach lowered regulatory barriers, streamlined the institutional review board (IRB) approval process at sites, and enabled sites to contribute to the analyses with minimal resources. Using this approach, we obtained a broad data-driven view of PASC across different countries, healthcare systems, patient populations, and time periods, and systematically examined all medical conditions across the different comparison groups. Central to our consortium effort is the ability of each local site to perform quality control by its own data scientists and clinicians. Other consortia, including Observational Health Data Sciences and Informatics (OHDSI) and Patient-Centered Clinical Research Network (PCORNet), have had similar success with federated EHR data networks^24,25^. A tradeoff for a large number of participating sites is the more limited ability to perform complex analyses. This contrasts with single data repositories such as the National COVID Cohort Collaborative^26^.

Our results indicate a possible high burden of long-term sequelae in patients recovering from SARS-CoV-2 infection. We observed a wide spectrum of PASC-related conditions not only in inpatient COVID-19 cases but also in outpatient cases. This supports the emerging evidence that even patients who did not experience severe disease requiring hospitalization during the acute period may experience long-term complications^27,28^. The similar PASC profiles between both the inpatient and outpatient COVID-19 cohorts suggest common underlying etiologic pathways in the development of PASC. We identified general symptoms that persist after initial infection, including malaise and fatigue, respiratory abnormalities, dysphagia, and loss of smell and taste, all of which are consistent with what is reported in the literature^8,29,30^. We additionally observed increased incidences of organ-specific dysfunction among patients with COVID-19, primarily involving dysfunction of the lungs, heart, and brain. Possible explanations for our findings include previously undiagnosed chronic conditions, adverse effects from treatments for SARS-CoV-2, and dysregulated inflammatory or hypercoagulable responses arising from SARS-CoV-2 infection^31,32^.

We observed that outpatient COVID-19 cases were at higher risk for thromboembolic events compared to controls, including both pulmonary embolism and venous thromboembolism. While there have been observational studies reporting high incidences of pulmonary embolisms in COVID-19 patients, most of these studies lacked appropriate control groups^33,34^. Interestingly, a recent study of 74,418 patients from 62 healthcare institutions reported a ninefold increased risk of pulmonary embolism among patients presenting to the emergency department with COVID-19-related pneumonia when compared to non-COVID-19 patients^35^. Moreover, venous thromboembolism incidence of up to 20% has been reported in COVID-19 inpatients, although again, the lack of appropriate inpatient controls limits the interpretation of these data^36^. Thus, our study confirms prior observational data that COVID-19 may be associated with an increased risk of thromboembolic events compared to non-COVID-19 patients in the outpatient setting. Unexpectedly, we did not find any significant associations of pulmonary embolism or venous thromboembolism in the COVID-19 inpatient group. One possible reason may be the use of prophylactic anticoagulation in the inpatient setting^37,38^. While these results may suggest a possible role for anticoagulation in patients with mild COVID-19 symptoms, a recent trial did not demonstrate any clinical benefit of anticoagulation or antiplatelet therapy in this population^39^.

Our results support emerging evidence that patients hospitalized with COVID-19 may be at increased risk for cardiac conditions including heart failure. Acute myocardial injury and elevated cardiac serum biomarker levels have been observed in COVID-19 patients and associated with severe COVID-19 and worse outcomes^40–44^. Prior observational cohort studies have reported new-onset heart failure in patients admitted with COVID-19-related pneumonia, including in patients with no prior history of congestive cardiac failure^45–47^. It is plausible that a new diagnosis of congestive cardiac failure in the post-acute period could suggest cardiomyopathy from systemic inflammatory responses in the setting of SARS-CoV-2 infection, direct SARS-CoV-2 myocardial infarction leading to myocarditis and eventual cardiac fibrosis, or as sequelae of severe COVID-19 predisposed by underlying cardiovascular comorbidities^47–53^. Furthermore, pulmonary hypertension and mechanical ventilation in COVID-19 patients with acute respiratory distress syndrome could contribute to right ventricular strain and decompensated heart failure in the long term^54–57^. Consistent with prior reports of subclinical myocardial injury who have recovered from recent COVID-19, we found higher incidences of angina pectoris and cardiac arrhythmias in inpatient and outpatient COVID-19 patients compared to controls^58^. These findings support emerging pathological studies that observed increased intramyocardial microthrombi in COVID-19 patients with ST-elevation myocardial infarction compared to controls^59^.

Among the neurological sequelae of COVID-19 patients, we noted consistent associations of increased risk of cognitive dysfunction and malaise in both COVID-19 inpatient and outpatient cohorts. Previous studies have hypothesized that cognitive dysfunction could be due to several reasons, including severe systemic inflammation, neuroinflammation, or complications of chronic illnesses during acute COVID-19^60–62^. Our observation of increased incidence of cognitive dysfunction, as well as malaise and fatigue, could be consistent with a myalgic encephalitis-like syndrome that have been proposed in prior reports of patients with post-acute sequelae^63,64^. While we also observed an increased risk for dementia, we should interpret this finding with caution given the typical long duration for the development of neurodegenerative conditions.

We observed changes in the incidence of sequelae in the inpatient and outpatient COVID-19 cohorts across ~15 months of the pandemic from early 2020 to early 2021. While the findings of decreasing incidence of cardiovascular and pulmonary conditions in the inpatient COVID-19 cohort may suggest improved patient management, this interpretation warrants caution and further validation. Interestingly, the incidence of metabolic conditions and sensory dysfunction (i.e., disorders of smell and taste) increased over time in both the inpatient and outpatient cohorts. While this could be due to changes in COVID-19 pathophysiology, an alternative explanation is that clinicians started to screen and document such conditions more systematically over time. Finally, in contrast to previous literature, we did not observe any significant changes over time in gastrointestinal or dermatological PASC phenotypes^19^. Further studies accounting for viral variants and administration of vaccines are needed to study trends in PASC incidence and mortality over different waves of the pandemic.

While the inpatient COVID-19 cases appeared to develop these new conditions after their positive SARS-CoV-2 polymerase chain reaction (PCR) test, these observations may be due to confounding and other types of bias. Compared to inpatient controls, the inpatient COVID-19 cases had worse preexisting health as evidenced by a higher baseline prevalence of pulmonary conditions, heart failure, chronic kidney disease, type 2 diabetes, and obesity. This cohort was also likely sicker on average compared to the inpatient controls, as they had a higher incidence of acute kidney injury and hypovolemia within the first 29 days of the index date. Outpatient COVID-19 cases had fewer preexisting comorbidities, i.e., only a higher prevalence of obesity and depression than outpatient controls.

This study has numerous limitations. First, we included only patients who were tested for SARS-CoV-2 in participating healthcare systems. As we were unable to ascertain the indications for hospital admission or SARS-CoV-2 testing, we could not completely mitigate selection bias or misclassification bias in cohort identification. While the inclusion of control cohorts is a major strength, we also could not ascertain the indication for control patients who were hospitalized or tested for SARS-CoV-2. Second, among the participating healthcare systems, only two non-U.S. sites could contribute control data. Third, given the limited scope of the common data capture and shared aggregate data, we could not control for patient-level potentially confounding variables such as comorbidities, medications, and other societal and environmental factors, all of which may induce bias. Accordingly, we were unable to stratify our analyses by demographic groups to further study PASC profiles. However, we note that risk ratio analyses were conducted using first occurrences of diagnosis codes, which better account for existing conditions among patients and make it more likely these are actually new diagnoses. Fourth, the study likely has several time-dependent biases: (1) not all patients had the same follow-up time in the study period, particularly in the late-stage period (90+ days after the index date); (2) we could not account for competing risks such as from death; (3) diagnosis codes may have been subject to censoring (transfer, discharge, death, and other loss to follow up) and thus dropout bias. Fifth, EHR data can have quality and completeness problems, especially for recent data, due to coding lag and pre-final codes. The degree to which this might have biased our analyses is likely the greatest in the final 2021-Q1 time period and depends on when individual hospitals ran their local database queries. Considering the aforementioned limitations, we caution against strong inferences from this study, which can identify associations and not identify mechanisms nor assess causality. In future studies, we plan to leverage patient-level EHR data to better mitigate many of these biases and investigate PASC profiles between patients of varying demographic groups.

## Methods

### Cohort identification

All patients who had a SARS-CoV-2 reverse transcription PCR test result recorded within the healthcare system were included in the data collection. COVID-19 patients were further classified as hospitalized (inpatient) or non-hospitalized (outpatient) based on whether or not they had a hospital admission between 7 days before or 14 days after a positive PCR test. If a patient had multiple positive PCR tests, the first positive PCR test was used. Inpatient COVID-19 cases’ index date was defined as the hospital admission date, and outpatient COVID-19 cases’ index date was defined as the date of the first positive PCR test.

Patients with one or more negative PCR tests, no positive PCR tests, and no U07.1 (“COVID-19, virus identified”) ICD-10 diagnosis codes were defined as controls. Controls were classified as inpatients or outpatients and index dates were defined in the same way as PCR-positive patients, according to the date of their first negative PCR test. There were 505,055 control inpatients and 1,825,473 control outpatients. Outpatients could include individuals who were later hospitalized after their index date, either for COVID-19 or unrelated conditions. We did not account for multiple hospitalizations in the inpatient cohort. We defined day zero as the index date.

### Federated data collection

Our analyses were performed on EHR data collected from 277 hospitals (affiliated with 17 regional healthcare systems) across five countries: France, Germany, Italy, Singapore, and the United States^20,65^. In the United States, we grouped the 170 Veterans Affairs hospitals into five regional healthcare systems^66^. See Table 2 for details of participating healthcare systems. The data cover information from January 1, 2020 to March 30, 2021; patient cohorts were additionally stratified by the calendar quarter of their index date to account for temporal changes in incidence, treatment, and SARS-CoV-2 variants, which of course were heterogeneous among the countries.

**Table 2:** Characteristics of participating healthcare systems.

Healthcare system	Country	Data collected on controls	Number of hospitals	Number of beds	Inpatient discharges per year
Assistance Publique—Hôpitaux De Paris	France	Yes	39	20,098	1,375,538
Beth Israel Deaconess Medical Center	USA	Yes	1	673	40,752
Bordeaux University Hospital	France	Yes	3	2676	130,033
ICSM Hospitals	Italy	No	3	775	12,344
Mass General Brigham (Partners Healthcare)	France	Yes	10	3418	163,521
National University Hospital	Singapore	No	1	1556	100,977
Policlinico Di Milano	Italy	No	1	900	40,000
University of California, LA	USA	Yes	2	786	40,526
University of Freiburg, Medical Center	Germany	No	1	1660	71,500
University of Kansas Medical Center	USA	No	1	794	54,659
University of Kentucky	USA	Yes	3	881	45,714
University of Michigan	USA	No	3	1000	49,008
University of Pittsburgh	USA	Yes	39	8085	369,300
VA North Atlantic	USA	Yes	49	3594	151,075
VA Southwest	USA	Yes	29	3115	156,315
Va Midwest	USA	Yes	39	2686	145,468
Va Continental	USA	Yes	24	2110	113,260
Va Pacific	USA	Yes	29	2296	114,569
		Totals	277	57,103	3,174,559

We distributed a SQL database script to contributing healthcare systems, which was manually run locally on EHR data to generate aggregate counts and statistics on patient cohorts after gaining local IRB approval^20,65,67^. The script was designed to run on clinical data repositories based on the Informatics for Integrating Biology & the Bedside (i2b2) data model, though several sites ported the code to their own data models if they did not use i2b2. Versions of the SQL script for both Microsoft SQL Server and Oracle databases are freely available on GitHub with an Apache 2.0 open source license^68^. Healthcare systems manually uploaded their aggregate result files to a central 4CE data upload website. Data collected included counts of patients, demographic characteristics, and truncated International Classification of Diseases (ICD) codes, Ninth or Tenth Revision, at three digits.

In order to ensure high-quality EHR data across countries, healthcare systems, and cohorts, multiple data quality control steps were performed. The 4CE data upload website ran an initial online quality control step, which checked that all files were under the standard format. This included the verification of the file and column names, column orders, data types, code values and ranges, and ensuring that there are no duplicated records. At the central site, additional quality control steps were completed on all submitted data. These steps included cross-validation consistency of the total case counts across all cohorts and verification of no negative values in patient counts. The central site also checked for consistency between the 3-digit ICD codes and the ICD dictionary. If a healthcare system presented any quality control issues, the central site directly contacted its corresponding informaticians to resolve them. These steps were crucial in ensuring proper downstream statistical analysis.

### Ethics approval

All study sites were responsible for and obtained ethics approval, as needed, from the appropriate ethics committee at their institution. The lead authors affirm that the manuscript is an honest, accurate, and transparent account of the study being reported; that no important aspects of the study have been omitted; and that any discrepancies from the study as originally planned have been explained. Approval was obtained at the Institutional Review Boards at Assistance Publique—Hôpitaux de Paris, Beth Israel Deaconess Medical Center, Bordeaux University Hospital, ICSM Hospitals, Mass General Brigham (Partners Healthcare), National University Hospital, Policlinico di Milano, University of Freiburg Medical Center, University of Kansas Medical Center, University of Kentucky, University of Pittsburgh, VA North Atlantic, VA Southwest, VA Midwest, VA Continental, and VA Pacific. The Institutional Review Boards at the University of California, Los Angeles and the University of Michigan made an exempt determination.

### Diagnosis code time periods and mapping

**Fig. 6:**
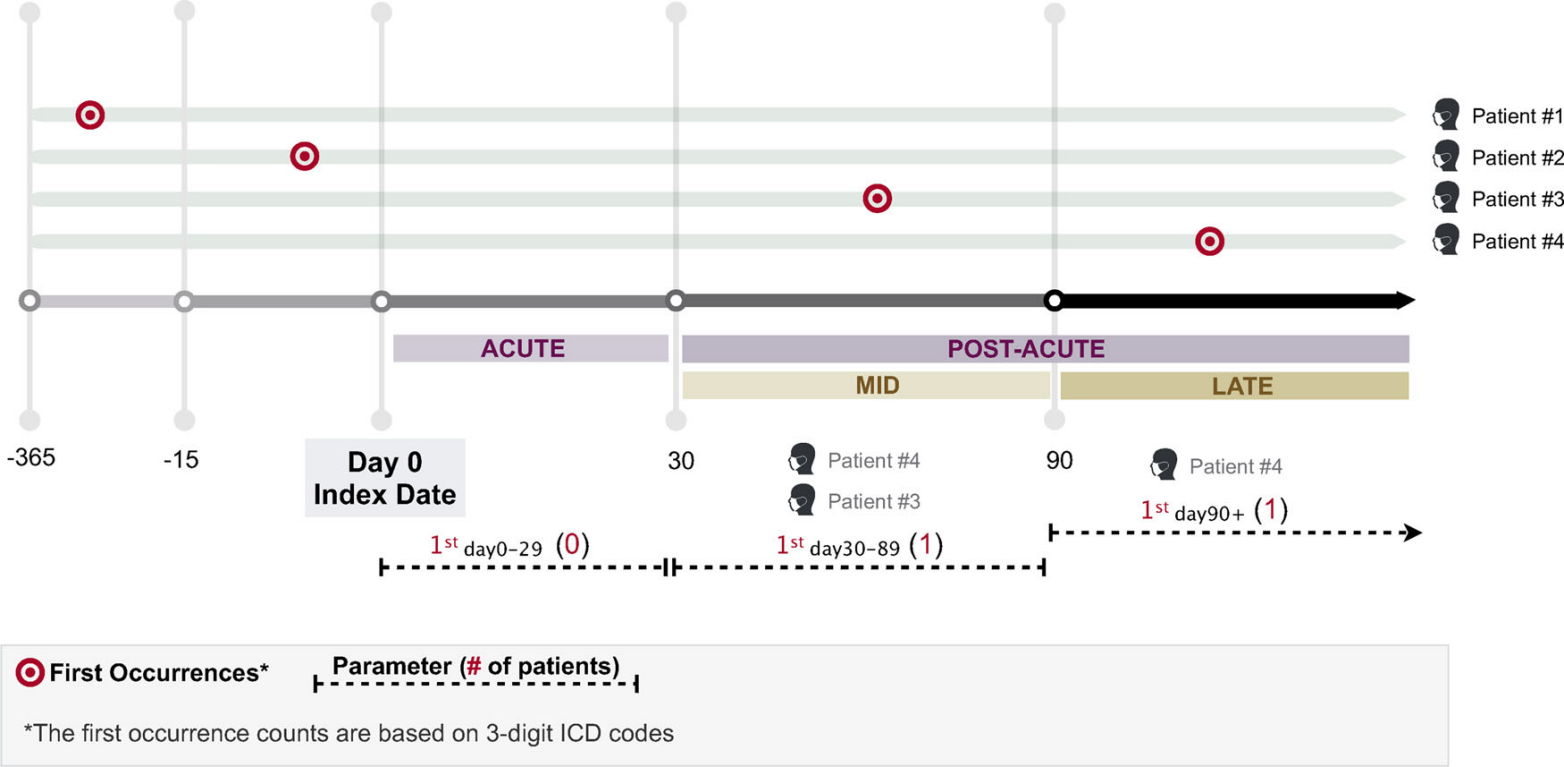
Study schematic of diagnosis code recording periods relative to the defined index date. Diagnosis codes in the post-acute period are defined as diagnosis codes recorded 30 days after initial infection. First occurrence diagnosis codes were defined as diagnosis codes which were not observed up to 365 days prior to the infection date.

Collected ICD code data were stratified into four time periods as follows: (1) recorded between 15 and 365 days prior to a patient’s index date; (2) recorded from 0 to 29 days after the index date (acute); (3) recorded from 30 to 89 days after the index date (mid-stage post-acute); and (4) recorded after 90 days from the index date (late-stage post-acute) (Fig. 6). We defined the first occurrence of an ICD code in a time period if there existed no prior annotations of the same ICD code in a patient’s EHR in preceding time periods. PheCodes were constructed by mapping ICD codes recorded in the EHRs to unique PheCodes following the standard procedure in ref. ^69^. Although healthcare systems in the United States use ICD-10 codes, some healthcare systems in other countries still use ICD-9. Mapping all ICD codes to PheCodes harmonized these differences.

### Statistical analysis

To account for heterogeneity between healthcare systems, DerSimonian and Laird random-effects meta-analyses were performed to aggregate individual healthcare system effect size estimates to produce an average effect size^70^. We summarized the prevalence of demographic subgroups between cohorts. We further summarized changes in demographic variable prevalence from 2020-Q1 to 2021-Q1. Fisher’s exact methods were used to estimate the prevalence confidence intervals.

The RR between cohorts of interest at specific time points were estimated within each healthcare system and then summarized across healthcare systems using a random-effects meta-analysis. Focusing on mid and late-stage post-acute periods, we estimated the RR of a phenotype in COVID-19 patients relative to control patients without COVID-19 as the ratio of the proportion of COVID-19 patients with an incident phenotype divided by the proportion of controls who have an incident phenotype. We further estimated the RR of a phenotype in inpatient COVID-19 cases relative to outpatient COVID-19 cases with the same approach as a proxy for disease severity, and we further normalized the risk ratio by dividing it by the risk ratio of a phenotype in inpatients without COVID-19 relative to outpatients without COVID-19. We denote this normalized risk ratio as relative RR. Statistical significance for risk ratios was defined as *P* < 0.05 after correction for multiple comparisons for an FDR of 5% using the Benjamini–Hochberg procedure^71^.

Additionally, as indicated in Weber et al., characteristics of patients with COVID-19 and risk for severe disease changed over the course of the pandemic^65^. Thus, we examined the incidence of conditions in the mid-stage period across calendar quarters. We defined the cumulative incidence during a specific time period as the proportion of patients with the first occurrence of an ICD code among all patients in the cohort.

All statistical analyses were performed using R software version 4.0.2.

### Reporting Summary

Further information on research design is available in the Nature Research Reporting Summary linked to this article.

## Supplementary information

Supplemental Material

Reporting Summary

## Data Availability

Only de-identified aggregate data was provided by sites for this study. We have implemented an online interactive visualization application in order to showcase the utility and diverse visualizations of the data at https://aggregate-pasc-4ce.herokuapp.com/.

## Code availability

The SQL database script that healthcare systems ran to generate the aggregate data is freely available on GitHub at https://github.com/covidclinical/PhaseX.2SqlDataExtraction. The R code that was used for the statistical analysis of this study is freely available on GitHub at https://github.com/covidclinical/Phase1.2PASCAnalysisRScript.
