# Supplementary material for: International electronic health record-derived post-acute sequelae profiles of COVID-19 patients
Source: NPJ Digit Med. 2022 Jun 29;5:81. doi: 10.1038/s41746-022-00623-8 (PMC9242995; doi:10.1038/s41746-022-00623-8)
Supplement: Supplementary file 1 — Supplemental Material [file 41746_2022_623_MOESM1_ESM.pdf]

Supplementary Information for

“International electronic health record-derived post-acute sequelae profiles of COVID-19 patients”

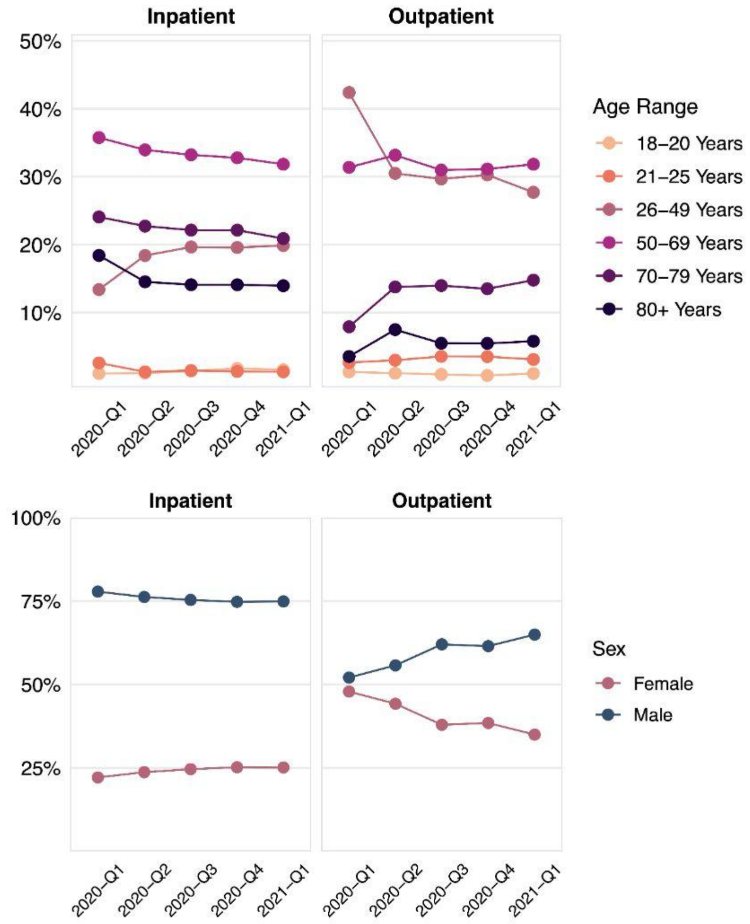

Supplementary Figure 1: Proportion of demographic characteristics for age and sex across time among control inpatients and outpatients.

Supplementary Note 1: Consortium for Clinical Characterization of COVID-19 by EHR (4CE) Members and Affiliations

James R Aaron[40], Giuseppe Agapito[41], Adem Albayrak[42], Giuseppe Albi[29], Mario Alessiani[43], Anna Alloni[38], Danilo F Amendola[44], François Angoulvant[45], Li L.L.J Anthony[46], Bruce J Aronow[33], Fatima Ashraf[47], Andrew Atz[48], Paul Avillach[1], Paula S Azevedo[49], James Balshi[50], Brett K Beaulieu-Jones[1], Douglas S Bell[24], Antonio Bellasi[51], Riccardo Bellazzi[29], Vincent Benoit[25], Michele Beraghi[52], José Luis Bernal-Sobrino[53], Mélodie Bernaux[54], Romain Bey[25], Surbhi Bhatnagar[33], Alvar Blanco-Martínez[53], Clara-Lea Bonzel[1], John Booth[55], Silvano Bosari[36], Florence T Bourgeois[12], Robert L Bradford[56], Gabriel A Brat[1], Stéphane Bréant[57], Nicholas W Brown[1], Raffaele Bruno[58], William A Bryant[55], Mauro Bucalo[38], Emily Bucholz[59], Anita Burgun[60], Tianxi Cai[1], Mario Cannataro[61], Aldo Carmona[62], Charlotte Caucheteux[63], Julien Champ[64], Jin Chen[65], Krista Y Chen[66], Luca Chiovato[32], Lorenzo Chiudinelli[67], Kelly Cho[30], James J Cimino[68], Tiago K Colicchio[68], Sylvie Cormont[57], Sébastien Cossin[31], Jean B Craig[69], Juan Luis Cruz-Bermúdez[53], Jaime Cruz-Rojo[53], Arianna Dagliati[2], Mohamad Daniar[70], Christel Daniel[71], Priyam Das[1], Batsal Devkota[72], Audrey Dionne[59], Rui Duan[3], Julien Dubiel[57], Scott L DuVall[73], Loic Esteve[74], Hossein Estiri[16], Shirley Fan[75], Robert W Follett[24], Thomas Ganslandt[76], Noelia García-Barrio[53], Lana X Garmire[77], Nils Gehlenborg[1], Emily J Getzen[78], Alon Geva[79], Tobias Gradinger[76], Alexandre Gramfort[63], Romain Griffier[31], Nicolas Griffon[71], Olivier Grisel[63], Alba Gutiérrez-Sacristán[1], Larry Han[3], David A Hanauer[9], Christian Haverkamp[80], Derek Y Hazard[81], Bing He[77], Darren W Henderson[40], Martin Hilka[57], Yuk-Lam Ho[23], John H Holmes[10,11], Chuan Hong[6,1], Kenneth M Huling[1], Meghan R Hutch[82], Richard W Issitt[55], Anne Sophie Jannot[83], Vianney Jouhet[31], Ramakanth Kavuluru[26], Mark S Keller[1], Chris J Kennedy[84], Daniel A Key[55], Katie Kirchoff[85], Jeffrey G Klann[16], Isaac S Kohane[1], Ian D Krantz[86], Detlef Kraska[87], Ashok K Krishnamurthy[88], Sehi L'Yi[1], Trang T Le[10], Judith Leblanc[89], Guillaume Lemaitre[63], Leslie Lenert[69], Damien Leprovost[90], Molei Liu[91], Ne Hooi Will Loh[92], Qi Long[93], Sara Lozano-Zahonero[21], Yuan Luo[82], Kristine E Lynch[73], Sadiqa Mahmood[42], Sarah E Maidlow[13], Adeline Makoudjou[21], Alberto Malovini[28], Kenneth D Mandl[66], Chengsheng Mao[82], Anupama Maram[94], Patricia Martel[95], Marcelo R Martins[96], Jayson S Marwaha[97], Aaron J Masino[98], Maria Mazzitelli[99], Arthur Mensch[100], Marianna Milano[101], Marcos F Minicucci[102], Bertrand Moal[14], Taha Mohseni Ahooyi[103], Jason H Moore[104], Cinta Moraleda[105], Jeffrey S Morris[106], Michele Morris[7], Karyn L Moshal[107], Sajad Mousavi[1], Danielle L Mowery[10], Douglas A Murad[24], Shawn N Murphy[15], Thomas P Naughton[108], Carlos Tadeu Breda Neto[44], Antoine Neuraz[17], Jane Newburger[59], Kee Yuan Ngiam[109], Wanjiku FM Njoroge[110], James B Norman[1], Jihad Obeid[69], Marina P Okoshi[102], Karen L Olson[111], Gilbert S. Omenn[20], Nina Orlova[57], Brian D Ostasiewski[112], Nathan P Palmer[1], Nicolas Paris[57], Lav P Patel[8], Miguel Pedrera-Jiménez[53], Emily R Pfaff[113], Ashley C Pfaff[114], Danielle Pillion[1], Sara Pizzimenti[36], Hans U Prokosch[115], Robson A Prudente[116], Andrea Prunotto[21], Víctor Quirós-González[53], Rachel B Ramoni[117], Maryna Raskin[42], Siegbert Rieg[118], Gustavo Roig-Domínguez[53], Pablo Rojo[119], Paula Rubio-Mayo[53], Paolo Sacchi[58], Carlos Sáez[120], Elisa Salamanca[57], Malarkodi Jebathilagam Samayamuthu[7], L. Nelson Sanchez-Pinto[121], Arnaud Sandrin[57], Nandhini Santhanam[76], Janaina C.C Santos[122], Fernando J Sanz Vidorreta[24], Maria Savino[123], Emily R Schriver[124], Petra Schubert[23], Juergen Schuettler[125], Luigia Scudeller[36], Neil J Sebire[55], Pablo Serrano-Balazote[53], Patricia Serre[57], Arnaud Serret-Larmande[126], Mohsin Shah[55], Zahra Shakeri Hossein Abad[1], Domenick Silvio[127], Piotr Sliz[66], Jiyeon Son[128], Charles Sonday[129], Andrew M South[39], Anastasia Spiridou[55], Zachary H. Strasser[16], Amelia LM Tan[1], Bryce W.Q. Tan[5], Byorn W.L. Tan[5], Suzana E Tanni[102], Deanne M Taylor[130], Ana I Terriza-Torres[53], Valentina Tibollo[28], Patric Tippmann[81], Emma MS Toh[34], Carlo Torti[99], Enrico M Trecarichi[99], Yi-Ju Tseng[131],

Andrew K Vallejos[132], Gael Varoquaux[133], Margaret E Vella[1], Guillaume Verdy[14], Jill-Jênn Vie[134], Shyam Visweswaran[7], Michele Vitacca[135], Kavishwar B Wagholikar[37], Lemuel R Waitman[136], Xuan Wang[1], Demian Wassermann[63], Griffin M Weber[1], Martin Wolkewitz[81], Scott Wong[5], Zongqi Xia[4], Xin Xiong[3], Ye Ye[7], Nadir Yehya[137], William Yuan[1], Alberto Zambelli[138], Harrison G Zhang[1], Daniela Zöller[21], Valentina Zuccaro[58], Chiara Zucco[101]

[1]Department of Biomedical Informatics, Harvard Medical School, Boston, United States, [2]Department of Electrical Computer and Biomedical Engineering, University of Pavia, Pavia, Italy, [3]Department of Biostatistics, Harvard T.H. Chan School of Public Health, Boston, United States, [4]Department of Neurology, University of Pittsburgh, Pittsburgh, United States, [5]Department of Medicine, National University Hospital, Singapore, Singapore, Singapore, [6]Department of Biostatistics and Bioinformatics, Duke University, Durham, United States, [7]Department of Biomedical Informatics, University of Pittsburgh, Pittsburgh, United States, [8]Department of Internal Medicine, Division of Medical Informatics, University Of Kansas Medical Center, Kansas City, United States, [9]Department of Learning Health Sciences, University of Michigan Medical School, Ann Arbor, United States, [10]Department of Biostatistics, Epidemiology, and Informatics, University of Pennsylvania Perelman School of Medicine, Philadelphia, United States, [11]Institute for Biomedical Informatics, University of Pennsylvania Perelman School of Medicine, Philadelphia, United States, [12]Department of Pediatrics, Harvard Medical School, Boston, United States, [13]Michigan Institute for Clinical and Health Research (MICHR) Informatics, University of Michigan, Ann Arbor, United States, [14]IAM unit, Bordeaux University Hospital, Bordeaux, France, [15]Department of Neurology, Massachusetts General Hospital, Boston, United States, [16]Department of Medicine, Massachusetts General Hospital, Boston, United States, [17]Department of biomedical informatics, Hôpital Necker-Enfants Malade, Assistance Publique Hôpitaux de Paris (APHP), University of Paris, Paris, France, [18]Department of Biomedical informatics, WiSDM, National University Health Systems Singapore, Singapore, Singapore, [19]Department of Anaesthesia, National University Health Systems Singapore, Singapore, Singapore, [20]Dept of Computational Medicine & Bioinformatics, Internal Medicine, Human Genetics, and School of Public Health, University of Michigan, Ann Arbor, United States, [21]Institute of Medical Biometry and Statistics, Faculty of Medicine and Medical Center, University of Freiburg, Freiburg, Germany, [22]Department of Ophthalmology, Mayo Clinic, Rochester, United States, [23]Massachusetts Veterans Epidemiology Research and Information Center (MAVERIC), VA Boston Healthcare System, Boston, United States, [24]Department of Medicine, David Geffen School of Medicine at UCLA, Los Angeles, United States, [25]IT Department, Innovation & Data, APHP Greater Paris University Hospital, Paris, France, [26]Division of Biomedical Informatics (Department of Internal Medicine), University of Kentucky, Lexington, United States, [27]Department of Preventive Medicine, Northwestern University, Chicago, USA, [28]Laboratory of Informatics and Systems Engineering for Clinical Research, Istituti Clinici Scientifici Maugeri SpA SB IRCCS, Pavia, Italy, [29]Department of Electrical, Computer and Biomedical Engineering, University of Pavia, Pavia, Italy, [30]Population Health and Data Science, MAVERIC, VA Boston Healthcare System, Boston, United States, [31]IAM unit, INSERM Bordeaux Population Health ERIAS TEAM, Bordeaux University Hospital / ERIAS - Inserm U1219 BPH, Bordeaux, France, [32]Unit of Internal Medicine and Endocrinology, Istituti Clinici Scientifici Maugeri SpA SB IRCCS, Pavia, Italy, [33]Departments of Biomedical Informatics, Pediatrics, Cincinnati Children's Hospital Medical Center, University of Cincinnati, Cincinnati, United States, [34]Yong Loo Lin School of Medicine, National University of Singapore, Singapore, Singapore, [35]Department of Medicine, National University Health Systems Singapore, Singapore, Singapore, [36]Scientific Direction, IRCCS Ca'

Granda Ospedale Maggiore Policlinico di Milano, Milan, Italy, [37]Department of Medicine, Massachusetts General Hospital, Boston, USA, [38]BIOMERIS (BIOMedical Research Informatics Solutions), Pavia, Italy, [39]Department of Pediatrics-Section of Nephrology, Brenner Children's, Wake Forest School of Medicine, Winston Salem, United States, [40]Department of Biomedical Informatics, University of Kentucky, Lexington, United States, [41]Department of Legal, Economic and Social Sciences, University Magna Graecia of Catanzaro, Catanzaro, Italy, [42]Health Catalyst, INC., Cambridge, United States, [43]Department of Surgery, ASST Pavia, Lombardia Region Health System, Pavia, Italy, [44]Clinical Research Unit of Botucatu Medical School, São Paulo State University, Clinical Research Unit of Botucatu Medical School, São Paulo State University, Botucatu, Brazil, [45]Pediatric emergency Department, Hôpital Necker-Enfants Malades, Assistance Public-Hôpitaux de Paris, Paris, France, [46]National Center for Infectious Diseases, Tan Tock Seng Hospital, Singapore, Singapore, [47]BIG-ARC, The University of Texas Health Science Center at Houston, School of Biomedical Informatics, Houston, United States, [48]Department of Pediatrics, Medical University of South Carolina, Charleston, United States, [49]Internal Medicine Department, Botucatu Medical School, São Paulo State University, Botucatu, Brazil, [50]Department of Surgery, St. Luke's University Health Network, Bethlehem, United States, [51]Department of Medicine, Division of Nephrology, Ente Ospedaliero Cantonale, Lugano, Switzerland, [52]IT Department, ASST Pavia, Voghera, Italy, [53]Health Informatics, Hospital Universitario 12 de Octubre, Madrid, Spain, [54]Strategy and Transformation Department, APHP Greater Paris University Hospital, Paris, France, [55]Digital Research, Informatics and Virtual Environments (DRIVE), Great Ormond Street Hospital for Children, UK, London, United Kingdom, [56]North Carolina Translational and Clinical Sciences (NC TraCS) Institute, UNC Chapel Hill, Chapel Hill, United States, [57]IT department, Innovation & Data, APHP Greater Paris University Hospital, Paris, France, [58]Division of Infectious Diseases I, Fondazione I.R.C.C.S. Policlinico San Matteo, Pavia, Italy, [59]Department of Cardiology, Boston Children's Hospital, Harvard Medical School, Boston, United States, [60]Department of Biomedical Informatics, HEGP, APHP Greater Paris University Hospital, Paris, France, [61]Department of Medical and Surgical Sciences, Data Analytics Research Center, University Magna Graecia of Catanzaro, Catanzaro, Italy, [62]Department of Anesthesia, St. Luke's University Health Network, Bethlehem, United States, [63]Université Paris-Saclay, Inria, CEA, Palaiseau, France, [64]INRIA Sophia-Antipolis – ZENITH team, LIRMM, Montpellier, France, [65]Department of Internal Medicine, University of Kentucky, Lexington, United States, [66]Computational Health Informatics Program, Boston Children's Hospital, Boston, United States, [67]UOC Ricerca, Innovazione e Brand reputation, ASST Papa Giovanni XXIII, Bergamo, Bergamo, Italy, [68]Informatics Institute, University of Alabama at Birmingham, Birmingham, United States, [69]Biomedical Informatics Center, Medical University of South Carolina, Charleston, United States, [70]Clinical Research Informatics, Boston Children's Hospital, Boston, United States, [71]IT department, Innovation & Data (APHP), UMRS1142 (INSERM), APHP Greater Paris University Hospital, INSERM, Paris, France, [72]Department of Biomedical and Health Informatics, Children's Hospital of Philadelphia, Philadelphia, United States, [73]VA Informatics and Computing Infrastructure, VA Salt Lake City Health Care System, Salt Lake City, United States, [74]SED/SIERRA, Inria Centre de Paris, Paris, France, [75]Health Information Technology & Services, University of Michigan, Ann Arbor, United States, [76]Heinrich-Lanz-Center for Digital Health, University Medicine Mannheim, Heidelberg University, Mannheim, Germany, [77]Department of Computational Biology and Bioinformatics, University of Michigan, Ann Arbor, United States, [78]Biostatistics, Perelman School of Medicine at the University of Pennsylvania, Philadelphia, United States, [79]Department of Anesthesiology, Critical Care, and Pain Medicine and Computational Health Informatics Program, Boston Children's Hospital, Boston, United States, [80]Institute of

Digitalization in Medicine, Faculty of Medicine and Medical Center, University of Freiburg, Freiburg, Germany, [81]Institute of Medical Biometry and Statistics, Institute of Medical Biometry and Statistics, Medical Center, University of Freiburg, Freiburg, Germany, [82]Department of Preventive Medicine, Northwestern University, Chicago, United States, [83]Department of Biomedical Informatics, HEGP, APHP Greater Paris University Hospital, Paris, France, [84]Center for Precision Psychiatry, Massachusetts General Hospital, Boston, United States, [85]Medical University of South Carolina, Charleston, United States, [86]Department of Pediatrics, Division of Human Genetics, The Children's Hospital of Philadelphia and the Perelman School of Medicine at the University of Pennsylvania, Philadelphia, United States, [87]Center for Medical Information and Communication Technology, University Hospital Erlangen, Germany, [88]Renaissance Computing Institute/Department of Computer Science, University of North Carolina, Chapel Hill, Chapel Hill, United States, [89]Clinical Research Unit, Saint Antoine Hospital, APHP Greater Paris University Hospital, Paris, France, [90]Clevy.io, Paris, France, [91]Department of Biostatistics, Harvard T. H. Chan School of Public Health, Boston, United States, [92]Department of Anaesthesia, National University Health Systems, Singapore, Singapore, Singapore, [93]Department of Biostatistics, Epidemiology and Informatics, University of Pennsylvania Perelman School of Medicine, Philadelphia, United States, [94]Harvard Catalyst, Harvard Medical School, Boston, United States, [95]Clinical Research Unit, Paris Saclay, APHP Greater Paris University Hospital, Boulogne-Billancourt, France, [96]Medical Informatics Center, Hospital das Clínicas, Faculty of Medicine of Botucatu, Clinical Research Unit of Botucatu Medical School, São Paulo State University, Botucatu, Brazil, [97]Department of Surgery, Beth Israel Deaconess Medical Center, Boston, United States, [98]Department of Anesthesiology and Critical Care, Children's Hospital of Philadelphia, Philadelphia, United States, [99]Department of Medical and Surgical Sciences, Infectious and Tropical Disease Unit, University Magna Graecia of Catanzaro, Catanzaro, Italy, [100]ENS, PSL University, Paris, France, [101]Department of Medical and Surgical Sciences, University Magna Graecia of Catanzaro, Catanzaro, Italy, [102]Internal Medicine Department of Botucatu Medical School, São Paulo State University, Botucatu, Brazil, [103]Department of Biomedical Health Informatics, Children's Hospital of Philadelphia, Philadelphia, United States, [104]Department of Computational Biomedicine, Cedars-Sinai Medical Center, West Hollywood, United States, [105]Pediatric Infectious Disease Department, Hospital Universitario 12 de Octubre, Madrid, Spain, [106]Department of Biostatistics, Epidemiology, and Informatics, Institute for Biomedical Informatics,, University of Pennsylvania Perelman School of Medicine, Berwyn, United States, [107]Department of Infectious Diseases, Great Ormond Street Hospital for Children, UK, London, United Kingdom, [108]Harvard Catalyst | The Harvard Clinical and Translational Science Center, Harvard Medical School, Boston, United States, [109]Department of Biomedical informatics, WiSDM, National University Health System Singapore, Singapore, Singapore, [110]Department of Psychiatry, University of Pennsylvania Perelman School of Medicine, Philadelphia, United States, [111]Computational Health Informatics Program and Department of Pediatrics, Boston Children's Hospital, Harvard Medical School, Boston, United States, [112]CTSI, WFBMI, Wake Forest School of Medicine, Winston Salem, United States, [113]NC TraCS Institute, UNC Chapel Hill, Chapel Hill, United States, [114]Department of Surgery, Beth Israel Deaconess Medical Center, Harvard Medical School, Boston, United States, [115]Department of Medical Informatics, University of Erlangen-Nürnberg, Erlangen, Germany, [116]Clinical Research Unit São Paulo State University, Brazil, Clinical Research Unit of Botucatu Medical School, São Paulo State University, Botucatu, Brazil, [117]Office of Research and Development, Department of Veterans Affairs, Washington, DC, United States, [118]Division of Infectious Diseases, Department of Medicine II, Medical Center – University of Freiburg, Faculty of Medicine, Freiburg, Germany, [119]Pediatric Infectious Disease Deparment, Hospital

Universitario 12 de Octubre, Madrid, Spain, [120]Biomedical Data Science Lab, ITACA Institute, Universitat Politècnica de València, Spain, Valencia, Spain, [121]Department of Pediatrics (Critical Care), Northwestern University Feinberg School of Medicine, Chicago, United States, [122]Nurse departament of FMB - medicine school of Botucatu, Clinical Research Unit of Botucatu Medical School, São Paulo State University, Botucatu, Brazil, [123]ASST Pavia, Lombardia Region Health System, Management Engineer, Direction, Pavia, Italy, [124]Data Analytics Center, University of Pennsylvania Health System, Philadelphia, United States, [125]Department of Anesthesiology, University Hospital Erlangen, FAU Erlangen-Nürnberg, Erlangen, Germany, [126]Hôpital Saint Louis, Department of Biostatistics and Bioinformatics, APHP Greater Paris University Hospital, Paris, France, [127]MICHR Informatics, University of Michigan, Ann Arbor, United States, [128]Department of Neurology, University of Pittsburgh Medical Center, Pittsburgh, United States, [129]Critical Care Medicine, Department of Medicine, St. Luke's University Health Network, Bethlehem, United States, [130]Department of Biomedical Health Informatics and the Department of Pediatrics, The Children's Hospital of Philadelphia and the University of Pennsylvania Perelman Medical School, Philadelphia, United States, [131]Department of Information Management, National Central University, Taoyuan, Taiwan, [132]Clinical & Translational Science Institute, Medical College of Wisconsin, Milwaukee, United States, [133]Université Paris-Saclay, Inria, CEA, Montréal Neurological Institute, McGill University, Palaiseau, France, [134]SequeL, Inria Lille, Villeneuve-d'Ascq, France, [135]Respiratory Department, ICS S. Maugeri IRCCS Pavia Italy, Lumezzane (BS), ITALY, [136]Department of Health Management and Informatics, University of Missouri, Columbia, Columbia, United States, [137]Department of Anesthesiology and Critical Care Medicine, Children's Hospital of Philadelphia and University of Pennsylvania, Philadelphia, United States, [138]Department of Oncology, ASST Papa Giovanni XXIII, Bergamo, Bergamo, Italy.
