# Supplementary material for: International electronic health record-derived post-acute sequelae profiles of COVID-19 patients
Source: NPJ Digit Med. 2022 Jun 29;5:81. doi: 10.1038/s41746-022-00623-8 (PMC9242995; doi:10.1038/s41746-022-00623-8)
Supplement: Supplementary file 2 — Reporting Summary [file 41746_2022_623_MOESM2_ESM.pdf]

nature portfolio

Corresponding author(s): Griffin M Weber

Last updated by author(s): May 4, 2022

Reporting Summary

Nature Portfolio wishes to improve the reproducibility of the work that we publish. This form provides structure for consistency and transparency in reporting. For further information on Nature Portfolio policies, see our Editorial Policies and the Editorial Policy Checklist.

Statistics

For all statistical analyses, confirm that the following items are present in the figure legend, table legend, main text, or Methods section.

n/a	Confirmed	
☐	☒	The exact sample size (*n*) for each experimental group/condition, given as a discrete number and unit of measurement
☐	☒	A statement on whether measurements were taken from distinct samples or whether the same sample was measured repeatedly
☐	☒	The statistical test(s) used AND whether they are one- or two-sided<br>*Only common tests should be described solely by name; describe more complex techniques in the Methods section.*
☐	☒	A description of all covariates tested
☐	☒	A description of any assumptions or corrections, such as tests of normality and adjustment for multiple comparisons
☐	☒	A full description of the statistical parameters including central tendency (e.g. means) or other basic estimates (e.g. regression coefficient) AND variation (e.g. standard deviation) or associated estimates of uncertainty (e.g. confidence intervals)
☐	☒	For null hypothesis testing, the test statistic (e.g. *F*, *t*, *r*) with confidence intervals, effect sizes, degrees of freedom and *P* value noted<br>*Give P values as exact values whenever suitable.*
☐	☒	For Bayesian analysis, information on the choice of priors and Markov chain Monte Carlo settings
☐	☒	For hierarchical and complex designs, identification of the appropriate level for tests and full reporting of outcomes
☐	☒	Estimates of effect sizes (e.g. Cohen's *d*, Pearson's *r*), indicating how they were calculated

Our web collection on statistics for biologists contains articles on many of the points above.

Software and code

Policy information about availability of computer code

Data collection	Publicly available SQL code was used to collect data for this study and it is available on the 4CE consortium GitHub website (link in the data availability statement of the manuscript).
Data analysis	Publicly available R code was used to conduct statistical analyses for this study and it is available on the 4CE consortium GitHub website (link in the data availability statement of the manuscript).

For manuscripts utilizing custom algorithms or software that are central to the research but not yet described in published literature, software must be made available to editors and reviewers. We strongly encourage code deposition in a community repository (e.g. GitHub). See the Nature Portfolio guidelines for submitting code & software for further information.

Data

Policy information about availability of data

All manuscripts must include a data availability statement. This statement should provide the following information, where applicable:

- Accession codes, unique identifiers, or web links for publicly available datasets
- A description of any restrictions on data availability
- For clinical datasets or third party data, please ensure that the statement adheres to our policy

Only de-identified aggregate data was provided by sites for this study. We have implemented an online interactive visualization application in order to showcase the utility and diverse visualizations of the data at https://aggregate-pasc-4ce.herokuapp.com/. The SQL database script that healthcare systems ran to generate the aggregate data is freely available in GitHub at https://github.com/covidclinical/PhaseX.2SqlDataExtraction. The R code that was used for the statistical analysis of this study is freely available in GitHub at https://github.com/covidclinical/Phase1.2PASCAnalysisRScript.

Field-specific reporting

Please select the one below that is the best fit for your research. If you are not sure, read the appropriate sections before making your selection.

☒ Life sciences ☐ Behavioural & social sciences ☐ Ecological, evolutionary & environmental sciences

For a reference copy of the document with all sections, see nature.com/documents/nr-reporting-summary-flat.pdf

Life sciences study design

All studies must disclose on these points even when the disclosure is negative.

Sample size	414,602 COVID cases, and 2.3 million controls
Data exclusions	NA
Replication	Separate analyses were conducted for each healthcare system, then a meta-analaysis was performed to synthesize the results obtained from each healthcare system.
Randomization	NA
Blinding	NA

Reporting for specific materials, systems and methods

We require information from authors about some types of materials, experimental systems and methods used in many studies. Here, indicate whether each material, system or method listed is relevant to your study. If you are not sure if a list item applies to your research, read the appropriate section before selecting a response.

Materials & experimental systems

n/a	Involved in the study	
☒	☐	Antibodies
☒	☐	Eukaryotic cell lines
☒	☐	Palaeontology and archaeology
☒	☐	Animals and other organisms
☐	☒	Human research participants
☒	☐	Clinical data
☒	☐	Dual use research of concern

Methods

n/a	Involved in the study	
☒	☐	ChIP-seq
☒	☐	Flow cytometry
☒	☐	MRI-based neuroimaging

Human research participants

Policy information about studies involving human research participants

Population characteristics	All patients who are part of any of the 18 healthcare systems represented in this study and who received a COVID PCR test.
Recruitment	This is a retrospective cohort study, and as such, no recruitment was needed.
Ethics oversight	Institutional Review Board approval was obtained at Assistance Publique - Hôpitaux de Paris, Beth Israel Deaconess Medical Center, Bordeaux University Hospital, Instituti Instituti Clinici Scientifici Maugeri Hospitals, Massachusetts General Brigham, National University Hospital, Policlinico di Milano, Medical Center University of Freiburg, University of Kentucky, University of Pittsburgh, and VA North Atlantic, Southwest, Midwest, Continental and Pacific. An exempt determination was made by Institutional Review Boards at University of California Los Angeles and University of Michigan.

Note that full information on the approval of the study protocol must also be provided in the manuscript.
